# Functional materials based on molecules with hydrogen-bonding ability: applications to drug co-crystals and polymer complexes

**DOI:** 10.1098/rsos.180564

**Published:** 2018-06-27

**Authors:** Kristin M. Hutchins

**Affiliations:** Department of Chemistry and Biochemistry, Texas Tech University, Lubbock, TX 79409, USA

**Keywords:** hydrogen bonding, pharmaceuticals, co-crystal, polymer

## Abstract

The design, synthesis and property characterization of new functional materials has garnered interest in a variety of fields. Materials that are capable of recognizing and binding with small molecules have applications in sensing, sequestration, delivery and property modification. Specifically, recognition of pharmaceutical compounds is of interest in each of the aforementioned application areas. Numerous pharmaceutical compounds comprise functional groups that are capable of engaging in hydrogen-bonding interactions; thus, materials that are able to act as hydrogen-bond receptors are of significant interest for these applications. In this review, we highlight some crystalline and polymeric materials that recognize and engage in hydrogen-bonding interactions with pharmaceuticals or small biomolecules. Moreover, as pharmaceuticals often exhibit multiple hydrogen-bonding sites, many donor/acceptor molecules have been specifically designed to interact with the drug via such multiple-point hydrogen bonds. The formation of multiple hydrogen bonds not only increases the strength of the interaction but also affords unique hydrogen-bonded architectures.

## 1. Introduction

The field of organic materials chemistry is consistently seeking to design and synthesize materials with novel and useful properties and functions. A general approach that has proven successful involves deliberate selection of the atoms, bonds and intra- or intermolecular interactions that will comprise the final material [1–5]. Analogous to retrosynthetic analysis for preparation of a new molecule [6], a retrosynthetic approach to functional materials involves using molecular building blocks that will interact through known and reliable interactions in the resulting material. These interactions may include covalent or non-covalent bonds. Non-covalent forces encompass hydrogen bonds, halogen bonds, metal–ligand coordination, pi–pi interactions, cation/anion–pi interactions and van der Waals forces. Such non-covalent forces are reversible, inherently weaker than covalent bonds and ubiquitous in nature [7,8]. For example, non-covalent interactions are responsible for a variety of recognition processes such as enzyme–substrate binding and maintaining the structure of proteins [9–11].

Organic materials chemists frequently draw inspiration from these large, elegant biological structures when designing new functional materials. Specifically, the field of supramolecular chemistry focuses on constructing complex chemical systems using non-covalent interactions [12,13]. One type of non-covalent interaction, the hydrogen bond, is recognized as the force responsible for maintaining the structure of the DNA double helix via bonds between base pairs. A hydrogen bond occurs when a hydrogen atom attached to an electronegative atom (D) interacts with a second electronegative atom (A) to form D–H⋯A [14]. The hydrogen bond is primarily an electrostatic interaction between the donor (D) and acceptor (A) atoms [15]. Hydrogen bonds can occur between atoms within the same molecule (intramolecular) or between atoms in different molecules (intermolecular).

In addition to assembling large biological molecules, hydrogen bonding has been used to construct crystalline and polymeric materials that exhibit novel and responsive properties [16–19]. One such property is the capacity to sense or recognize other molecules, which often occurs due to an interaction between the two species, such as non-covalent forces [20,21]. This review will focus on crystalline and polymeric materials that can sense and interact with pharmaceutical compounds via formation of intermolecular hydrogen bonds. Utilization of hydrogen-bonding interactions to engage a pharmaceutical compound has proven effective in applications including drug property modification, sensing/recognition and drug delivery. Here, we will discuss the design strategies and successful applications of some of these materials.

## 2. The synthon approach

**Figure 1.**
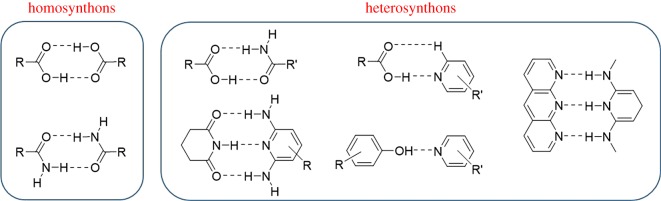
Examples of synthons based on hydrogen bonds: homosynthons and heterosynthons.

Successful formation of hydrogen bonds between two molecules involves designing appropriate donor and acceptor moieties that will engage in the interaction. A common approach is to use synthons, which are ‘structural units within a molecule that can be assembled using known synthetic operations’ [22]. In the field of supramolecular chemistry, these synthons typically exist as homosynthons or heterosynthons (figure 1). Homosynthons contain identical functional groups that exhibit molecular complementarity, and they are often deemed ‘self-association motifs’ [23]. Classic examples of homosynthons are dimers composed of carboxylic acids or amides. Conversely, heterosynthons contain non-identical functional groups, but they also exhibit molecular complementarity [23,24]. Examples of heterosynthons sustained by hydrogen bonds that range from strong to medium in strength are often based on O–H⋯N interactions [25] and include carboxylic acid⋯amide dimers, carboxylic acid⋯aromatic nitrogen and phenol⋯aromatic nitrogen. Weaker hydrogen bonds based on C–H⋯N and C–H⋯O interactions have also become common in the literature [25].

**Figure 2.**
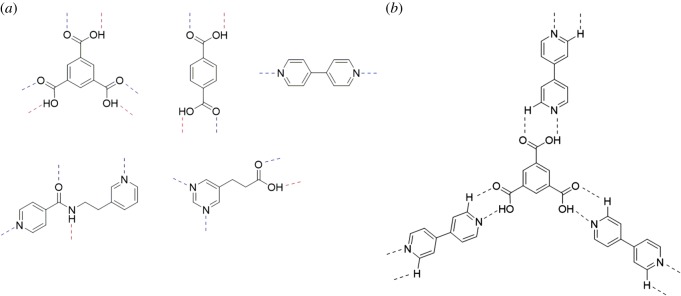
Examples of multitopic hydrogen bonding in: (*a*) donor and acceptor molecules and (*b*) co-crystal. Donor sites indicated with red dashed lines and acceptor sites with blue dashed lines.

While single- or two-point hydrogen-bonding interactions have proven highly effective in assembling donor and acceptor molecules, multitopic hydrogen-bond donors and acceptors have found significant use in designing materials with higher dimensionality (i.e. one dimensional (1D) to three dimensional). In this regard, the di- and tricarboxylic acid families [26], as well as aromatics with multiple donor/acceptor sites [27], are common (figure 2*a*). Furthermore, donor and acceptor molecules that exhibit both shape complementarity and multi-point hydrogen-bonding interactions have been used as an effective method to ensure recognition between two molecules [28–30].

## 3. Co-crystals involving pharmaceutical compounds

Co-crystals, which are crystalline solids composed of two or more neutral molecules typically in a stoichiometric ratio [31–33] (figure 2*b*), have garnered interest as a means to modify the properties of pharmaceutical compounds [34,35]. These properties can include mechanical, solubility, stability, bioavailability and additional physical properties [36–38]. The pharmaceutical co-crystal community is particularly interested in modifying properties of drugs that exhibit poor performance as single-component solids. Other crystalline forms of pharmaceuticals such as solvates, hydrates, salts and polymorphs have also been used to modify drug properties [39–43]. However, for the purpose of this review, neutral co-crystalline solids will be the primary focus.

Co-crystals involving pharmaceutical compounds are typically composed of the active pharmaceutical ingredient (API), and the second component is often on the US Food and Drug Administration's Generally Recognized as Safe (GRAS) list [34]. Using a compound on the GRAS list is advised if the goal is to implement the pharmaceutical co-crystal into clinical settings. However, many co-crystals involving APIs have been synthesized using compounds that are not on the GRAS list in order to better understand the synthon behaviour and properties of the API.

Pharmaceuticals that contain multiple hydrogen-bond donor and acceptor sites are especially interesting because there is flexibility in the synthons and second components that can be used for co-crystallization studies. Moreover, the synthon behaviour of such pharmaceuticals is intriguing because the same functional group can often form different hydrogen bonds, depending on the donor/acceptor molecule that is used. In this section, we will discuss the hydrogen-bonding behaviour of pharmaceutical compounds that possess multiple sites capable of engaging in these intermolecular interactions.

### 3.1. Pharmaceuticals with multiple hydrogen-bond-donor sites: co-crystals involving paracetamol and polyphenols

**Figure 3.**
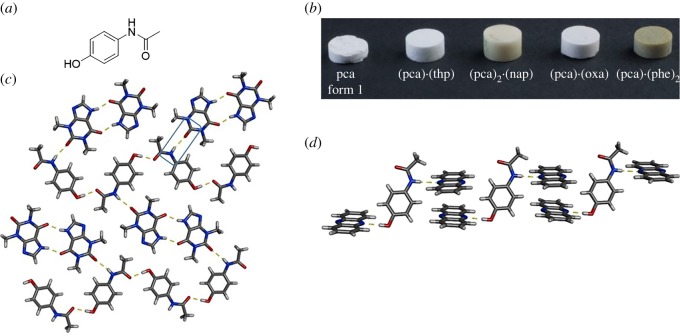
PCA: (*a*) chemical structure, (*b*) tablets of pure form and co-crystals, (*c*) X-ray crystal structure of PCA·THP highlighting the interaction between the two components and (*d*) X-ray crystal structure of PCA·2(PHE). Hydrogen bonds indicated with yellow dashed lines. (*b*) Reproduced with permission from Karki *et al*. [46]. Copyright © 2009 JohnWiley & Sons.

Paracetamol (PCA) is a well-known and widely utilized drug used to treat fever and pain. PCA commonly exists in two polymorphic forms, form I and II [44,45]. Form I is the thermodynamically stable form, but it displays poor compaction properties [36]. Form II exhibits superior compaction properties, likely due to the parallel arrangement of hydrogen-bonded layers in its crystalline form, but its lower thermodynamic stability (compared with form I) has prevented commercial use [46]. There have been significant efforts towards improving the compaction properties of PCA through co-crystallization [46–48], and many efforts have focused on achieving layered structures. PCA comprises two functional groups that can be used as synthon handles, a hydroxyl group and an amide group (figure 3*a*). Jones and co-workers demonstrated significant improvements in compaction properties in hydrogen-bonded co-crystals of PCA with theophylline (THP), oxalic acid (OXA) and phenazine (PHE) (figure 3*b*) [46]. Specifically, the co-crystal PCA·THP, which was first reported by Childs *et al*. [49], exhibited the largest improvement in tensile strength. The components of PCA·THP assemble to form a layered structure sustained by numerous hydrogen bonds. Neighbouring PCA molecules interact with each other via O–H⋯O hydrogen bonds, while adjacent THP molecules interact through N–H⋯O hydrogen-bonded dimers (figure 3*c*) [46]. PCA and THP interact with each other via a N–H(PCA)⋯O(THP) heterosynthon. The hydrogen-bonding motifs in the co-crystal PCA·2(PHE), however, are much different because both hydrogen-bond-donor groups of PCA interact with PHE. The assembly of the two molecules in the co-crystal is facilitated by O–H⋯N and N–H⋯N hydrogen bonds between PCA and PHE, resulting in Z-shaped assemblies with stacked PHE molecules (figure 3*d*) [46].

**Figure 4.**
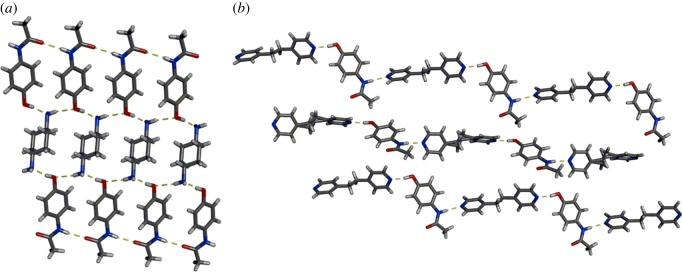
X-ray crystal structures of: (*a*) 2(PCA)·1,4AC and (*b*) PCA·BPEth. Hydrogen bonds indicated with yellow dashed lines.

The ability to form layered structures in co-crystals involving PCA was also reported by Powell and co-workers [50]. The second components used for co-crystallization were dual-hydrogen-bond acceptor molecules; however, the two donors of PCA did not always interact with both acceptor sites. In a co-crystal of PCA and *trans*-1,4-diaminocyclohexane (1,4AC), the hydroxyl groups of PCA form hydrogen bonds with the amino groups of 1,4AC (figure 4*a*). The amide groups of neighbouring PCA molecules assemble via N–H⋯O hydrogen bonds to form infinite chains. Conversely, in co-crystals of PCA and 1,2-bis(4-pyridyl)ethane (BPEth), both hydrogen-bond donating groups of PCA interact with the aromatic nitrogen atoms of BPEth to form infinite 1D chains (figure 4*b*) [50]. In both co-crystals, the 1D hydrogen-bonded chains stack to form layered structures. Additional cases of synthon diversity with PCA have been demonstrated in other co-crystals and numerous hydrate and solvate structures [51–56].

**Figure 5.**
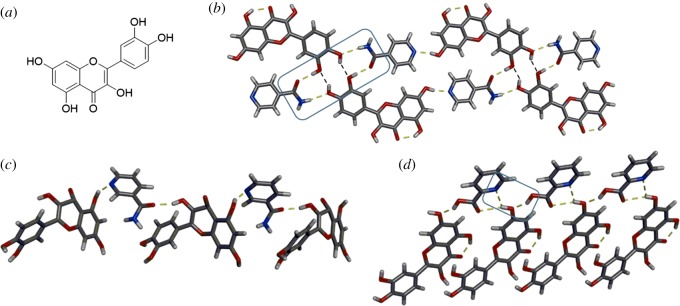
QUE: (*a*) chemical structure, and X-ray crystal structures of: (*b*) QUE·INM highlighting the four-component supramolecular assembly (homosynthons indicated with black dashed lines), (*c*) QUE·NICO and (*d*) QUE·PICO highlighting bifurcated hydrogen bonding. Hydrogen bonds indicated with yellow dashed lines.

Quercetin (QUE) is a polyphenol found in fruits and vegetables. Polyphenols are often used in dietary supplements and studied for their antioxidant activity. QUE exhibits poor water solubility and is susceptible to metabolic conjugation, which contributes to its low bioavailability [57,58]. QUE has numerous hydrogen-bond-donor sites (figure 5*a*), which makes the molecule an ideal candidate for co-crystallization studies. Moreover, the multitude of sites and conformational flexibility of QUE offer the opportunity to study the variety of synthons that could form in multi-component crystals. Zaworotko, Shytle and co-workers reported improved solubility and bioavailability of QUE in co-crystals with isonicotinamide (INM) and caffeine (CAF) [59]. The two molecules in the QUE·INM co-crystal assemble to form a four-component assembly sustained by six hydrogen bonds. Homosynthons based on O–H⋯O interactions between QUE molecules and N–H⋯O and O–H⋯O heterosynthons between QUE and INM give rise to the supramolecular assembly (figure 5*b*). The crystal structure of QUE·CAF was not determined, but it was determined to be isostructural to a solvated structure of QUE·CAF·methanol [59]. A 14-fold increase in the solubility of QUE was achieved with the QUE·CAF co-crystal.

Vasisht and co-workers recently reported the co-crystallization of QUE with two GRAS list compounds, nicotinamide (NICO) and picolinic acid (PICO), via liquid-assisted grinding [60]. In the QUE·NICO co-crystal, the aromatic nitrogen and carbonyl of NICO both act as hydrogen-bond acceptors and the hydroxyl groups of QUE serve as hydrogen-bond donors (figure 5*c*). The QUE·PICO co-crystal exhibits slightly different hydrogen-bonding behaviour. One hydroxyl group of QUE forms bifurcated hydrogen bonds with the aromatic nitrogen of one PICO molecule and the carboxylic acid of a second PICO molecule (figure 5*d*). In both co-crystals, the remaining hydroxyl groups of QUE interact with neighbouring QUE molecules through O–H⋯O homosynthons. Both co-crystals exhibited increased dissolution rates, higher solubility and enhanced antioxidant and antihaemolytic activity when compared with QUE as a single-component solid [60].

**Figure 6.**
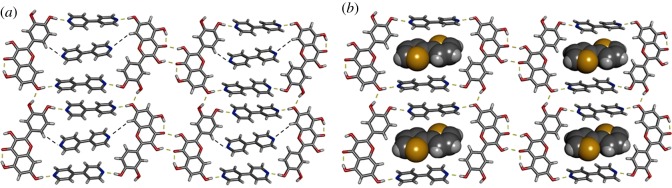
X-ray crystal structures involving QUE: (*a*) QUE·4,4′BP 2109 (*b*) QUE·4,4′BP·2,2′TP ternary co-crystal. Hydrogen bonds indicated with yellow dashed lines. C–H⋯N interactions indicated with black dashed lines.

Dubey and Desiraju also recently examined the co-crystal landscape of QUE via a combinatorial approach [61]. A variety of dihydrogen-bond-acceptor molecules were used and co-crystals, solvates and polymorphs were successfully synthesized. An example of a co-crystal with 4,4′-bipyridine (4,4′BP) is shown in figure 6*a*. The assembly of QUE·4,4′BP involves two molecules of QUE forming four O–H⋯N hydrogen bonds to two 4,4′BP molecules. A third 4,4′BP is sandwiched within the cavity via C–H⋯N hydrogen bonds. Most notably, however, was the ability to synthesize novel ternary (three component) co-crystals involving QUE [61]. When QUE, 4,4′BP, and 2,2′-bithiophene (2,2′TP) were co-crystallized, a similar structure was obtained; however, the 2,2′TP replaced the third 4,4′-BP in the cavity (figure 6*b*).

**Figure 7.**
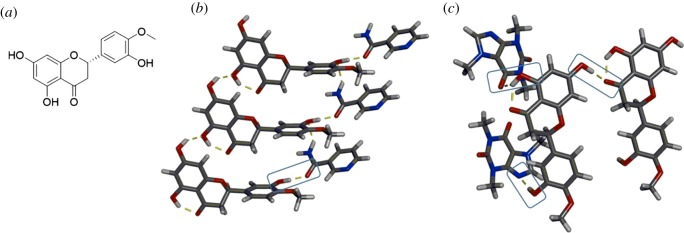
Structures involving HESP: (*a*) chemical structure, (*b*) X-ray structure of HESP·NICO highlighting one heterosynthon and (*c*) X-ray structure of HESP·CAF highlighting the three different synthons formed with hydroxyl groups. Hydrogen bonds indicated with yellow dashed lines.

Another polyphenol that can act as an antioxidant and exhibits structural similarity to QUE is hesperetin (HESP). HESP has three hydroxyl groups that can act as hydrogen-bond donor sites and a carbonyl that can act as a hydrogen-bond acceptor (figure 7*a*) [62]. Vasisht and co-workers recently reported co-crystallization of HESP with NICO and CAF and observed different synthon behaviour [63]. In the HESP·NICO co-crystal, only one of the three hydroxyl groups of HESP engages in a hydrogen bond with NICO via a O–H⋯O heterosynthon involving the carbonyl. The other two hydroxyl groups of HESP interact via O–H⋯O homosynthons to form a 1D chain (figure 7*b*). On the other hand, two of three hydroxyl groups of HESP engage in heterosynthons in the co-crystal of HESP·CAF (figure 7*c*). These two hydroxyl groups interact with CAF via O–H⋯O and O–H⋯N heterosynthons involving the carbonyl and aromatic nitrogen of CAF, respectively. The third hydroxyl group of HESP engages in an O–H⋯O homosynthon with the carbonyl of a neighbouring HESP moiety. Interestingly, when HESP is co-crystallized in the presence of similar hydrogen-bond acceptor motifs, the same synthons do not pervade [63].

### 3.2. Pharmaceuticals with hydrogen-bond donor and acceptor sites: co-crystals involving anti-tuberculosis and diuretic drugs

For molecules possessing multiple hydrogen-bond donor and acceptor sites, prediction of all possible synthons that can form is challenging. Etter's rules regarding best donor–best acceptor pairing certainly aid in this prediction ability [64]. However, structural characterization of these types of molecules in multi-component crystals will generate synthon behaviour data that can be used to achieve successful synthon design and formation.

**Figure 8.**
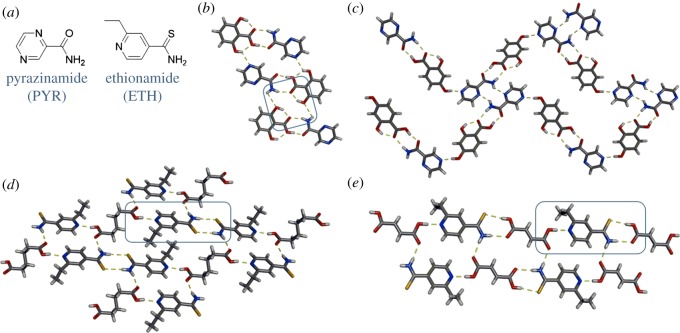
Structures of: (*a*) APIs PYR and ETH, and X-ray structures of: (*b*) PYR·2,6-dihydroxybenzoic acid highlighting the ring motif, (*c*) PYR·2,4-dihydroxybenzoic acid with the chain motif, (*d*) ETH·ADP highlighting hetero- and homosynthons, and (*e*) ETH·FA highlighting two heterosynthons. Hydrogen bonds indicated with yellow dashed lines.

The anti-tuberculosis pharmaceuticals pyrazinamide (PYR) and ethionamide (ETH) are structurally similar and comprise both hydrogen-bond donor and acceptor sites. The ability of the drugs to form multiple hydrogen bonds [65,66] makes them synthetically and structurally intriguing. The API PYR contains an amide and two aromatic nitrogen atoms as synthon handles (figure 8*a*), and is frequently used in combination with other drugs, which can pose compatibility and bioavailability issues [65]. There have been 33 co-crystal structures of PYR with a unique second component reported, and 31 of the 33 involve hydrogen bonding [67], indicating the frequency of its use as a synthetic tool. Jarzembska, Woźniak and co-workers recently described a series of co-crystals with PYR and a variety of dihydroxybenzoic acids [68]. In all cases, the carboxylic acid⋯amide heterosynthon between PYR and the acid formed. However, with the presence of additional donor (hydroxyl) and acceptor (nitrogen) moieties in the two components, supplementary hydrogen-bonding interactions comprise the extended structure. The specific interactions depend on the substitution of the hydroxyl groups on the benzoic acid. In the co-crystal involving PYR and 2,6-dihydroxybenzoic acid, ring motifs are generated (figure 8*b*), whereas co-crystallization of PYR with 2,4-dihydroxybenzoic acid affords chain motifs (figure 8*c*). Mei and co-workers observed similar carboxylic acid⋯amide heterosynthon dimers in co-crystals of PYR with *trans*-aconitic acid and citric acid [66]. Superior solubility and intrinsic dissolution rates were realized with both co-crystals when compared with PYR as a single-component solid.

The drug ethionamide (ETH) is structurally similar to PYR and is also used as an anti-tuberculosis medication. ETH contains an aromatic nitrogen atom and a thioamide functional group (figure 8*a*), and exhibits poor aqueous solubility. Nangia and co-workers synthesized co-crystals of ETH with dicarboxylic acids and observed different synthon formation when the length and saturation of the diacid was changed [69]. Co-crystals of ETH and the saturated diacids adipic acid (ADP) and suberic acid are sustained by a combination of hydrogen bonds based on heterosynthons and homosynthons. For example, in the ETH·ADP co-crystal, the two molecules interact with each other via the acid⋯pyridine heterosynthon, while neighbouring ETH molecules interact via thioamide⋯thioamide dimer homosynthons (figure 8*d*). However, the co-crystal involving ETH and the unsaturated diacid fumaric acid (FA) is sustained by two heterosynthons based on acid⋯pyridine and acid⋯thioamide hydrogen bonds (figure 8*e*). Thus, although the same functional groups are used in the co-crystals, different supramolecular interactions dominate the resulting structures. The dissolution rate of ETH was improved in all co-crystals, and a salt composed of ETH and oxalic acid exhibited the highest bioavailability enhancement [69].

**Figure 9.**
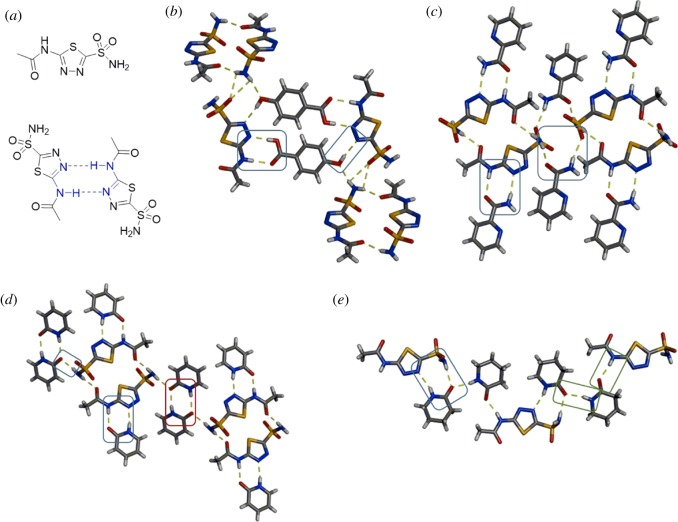
Structures involving ACZ: (*a*) chemical structure of API and the self-association motif. X-ray crystal structures of: (*b*) ACZ·4HBA highlighting the acid and hydroxyl interactions, (*c*) ACZ·2PAM highlighting the amide interactions, (*d*) ACZ·2(2HP) with heterosynthons (blue) and homosynthons (red) denoted and (*e*) ACZ·2(VLM) with the two types of amide interactions highlighted (blue and green). Hydrogen bonds indicated with yellow dashed lines.

The API acetazolamide (ACZ) is a diuretic and carbonic anhydrase inhibitor used to treat a variety of conditions including glaucoma, epilepsy and altitude sickness. ACZ contains a sulfonamide, a thiadiazole and an amide as functional groups (figure 9*a*). Even when ACZ is incorporated into multi-component crystals, the amide NH and thiadiazole of ACZ often self-associate to form dimers [70,71] (figure 9*a*). The hydrogen-bonding behaviour of ACZ makes the design of co-crystals wherein the two components will interact with each other particularly challenging. Herrera-Ruiz, Höpfl and co-workers demonstrated successful co-crystallization of ACZ using 4-hydroxybenzoic acid (4HBA) and 2-pyridinecarboxamide (2PAM) as the second components [70,72]. In the co-crystal of ACZ·4HBA, both the benzoic acid and hydroxyl groups of 4HBA interact with ACZ. The benzoic acid participates in O–H⋯N and N–H⋯O heterosynthon dimers with the thiadiazole nitrogen and amide NH (figure 9*b*). The hydroxyl group forms an O–H⋯N heterosynthon with the other nitrogen of the thiadiazole. When 2PAM was used as the second component, co-crystals of ACZ·2PAM sustained by heterosynthon dimers involving the thiadiazole nitrogen and amide NH were also achieved (figure 9*c*). A second molecule of 2PAM forms two N–H⋯O hydrogen-bonded heterosynthons with the sulfonamide portion of ACZ.

Bolla and Nangia have also demonstrated successful co-crystallization of ACZ using a variety of cyclic amide motifs [71]. When 2-pyridone (2HP) was used as the second component, a co-crystal of ACZ·2(2HP) was obtained. One 2HP molecule engages with ACZ via N–H⋯N and N–H⋯O heterosynthon dimers, similar to the co-crystal of ACZ·2PAM (figure 9*d*). The second unique 2HP forms homosynthon amide dimers with itself and connects the 2HP-ACZ units via N–H⋯O heterosynthons. The saturated, cyclic amide valerolactam (or 2-piperidinone, VLM) also co-crystallized with ACZ in a 2:1 ratio to form ACZ·2(VLM). One VLM molecule interacts with ACZ via N–H⋯N and N–H⋯O heterosynthons involving the thiadiazole nitrogen and sulfonamide NH (figure 9*e*). The second molecule of VLM bridges the VLM-ACZ units via N–H⋯O hydrogen bonds. The first ternary co-crystal of ACZ was also synthesized using two amides, 2HP and NICO [71].

## 4. Recognition of small molecules by polymer materials

Polymers that are capable of recognizing other molecules have found applications in sensing, drug delivery and separations. Like crystalline materials, these polymers rely on a recognition event between the polymer and the second species (e.g. drug). This recognition is often based on non-covalent interactions such as van der Waals forces, electrostatic, π-interactions, or hydrophobic effects [21]. For recognition based on hydrogen-bonding interactions, a variety of polymers have been used with molecular recognition strategies ranging from highly specific synthetic receptors to non-specific bonding to the polymer chain. Some common polymers used for non-specific hydrogen-bonding interactions include polyethylene glycol (PEG) [73], polyvinylpyrrolidone (PVP) [74] and cellulose [75,76].

Similar to the previous section on crystalline materials, small molecules containing multiple hydrogen-bond donor and acceptor sites open the door to a variety of polymeric recognition motifs (specific and non-specific) that can be explored. In this section, we will discuss polymers using both types of recognition motifs to bind pharmaceuticals and small biomolecules for a variety of applications.

### 4.1. Design of polymers that bond with pharmaceutical compounds

**Figure 10.**
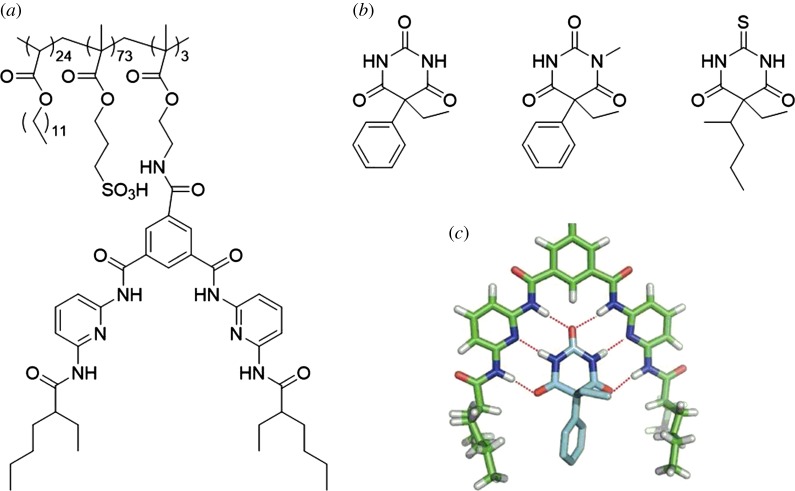
Barbiturate structures: (*a*) functionalized polymer receptor, (*b*) examples used in the study (left to right: phenobarbital, mephobarbital and thiopental) and (*c*) lowest energy conformation of phenobarbital bound to the receptor. (*c*) Reproduced with permission from Loizidou *et al.* [77]. Copyright © 2011 JohnWiley & Sons.

Incorporation of a specific receptor into a polymer backbone was demonstrated by Loizidou and co-workers wherein a barbiturate receptor was designed and synthesized (figure 10*a*) [77]. Barbiturates are central nervous system depressants and exhibit both hydrogen-bond donor (amine) and acceptor (carbonyl) sites, depending on the specific barbiturate molecule (figure 10*b*). The polymer receptor used in this study is multitopic, featuring hydrogen-bond donor and acceptor sites, and would be capable of forming multiple hydrogen bonds to the barbiturate molecules. The recognition ability of the polymer was tested in aqueous solutions of four barbiturates that comprise different numbers of hydrogen-bonding sites. Binding constants ranging from 10^2^ to 10^5^ M^−1^ were realized in solution, and computational studies revealed that a combination of hydrogen bonding and binding geometry contribute to the recognition (figure 10*c*) [77].

**Figure 11.**
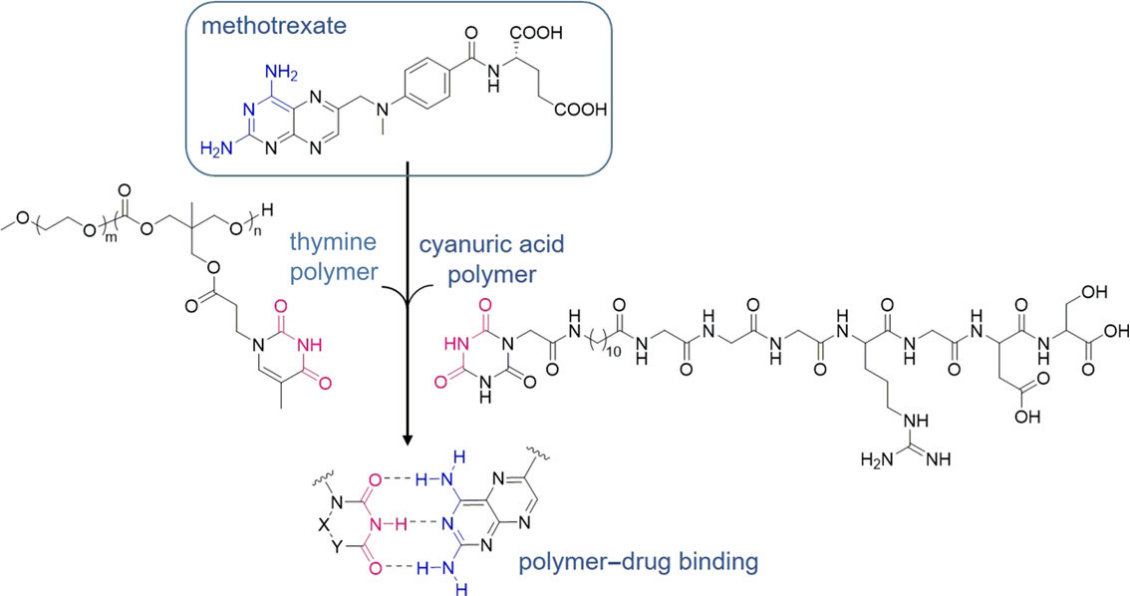
Chemical structures of MTX (blue), thymine-functionalized or cyanuric acid-functionalized polymers (pink), and hydrogen-bonded complex.

Another example of using a multitopic hydrogen-bonding polymer to bind a drug molecule was reported by He and co-workers [78]. A thymine-functionalized monomer was copolymerized with ethylene glycol to generate functional polymer micelles. The anti-cancer and immune system suppressant drug methotrexate (MTX) was successfully bound to the polymers via three-point hydrogen bonds between thymine and MTX (figure 11). The formation of hydrogen bonds between the polymer and drug was confirmed by nuclear magnetic resonance and infrared (IR) spectroscopy. Furthermore, due to the reversible nature of hydrogen bonds, the MTX was successfully released from the polymer micelles under acidic conditions.

A similar strategy was used by Xu, Zhang and co-workers where a cyanuric acid-functionalized polymer was designed and synthesized [79]. Although the polymer backbone is different, the multitopic hydrogen-bonding recognition motif is identical (figure 11). MTX was successfully recognized, loaded into the polymer and released under physiological or acidic conditions.

**Figure 12.**
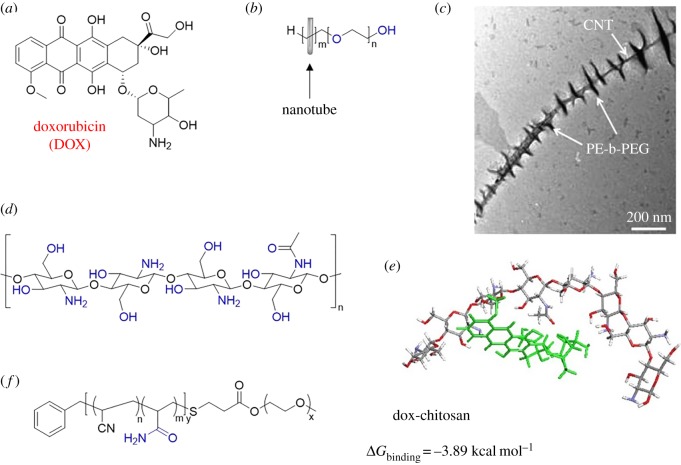
Materials used to bond with DOX: (*a*) chemical structure of DOX, (*b*) PE-b-PEG-functionalized hybrid material, (*c*) transmission electron microscopy image of PE-b-PEG-functionalized hybrid CNT material, (*d*) chitosan polymer, (*e*) simulation of DOX–chitosan binding and (*f*) (mPEG-b-P(AAm-co-AN)) copolymer. Hydrogen-bonding sites on polymers indicated in blue. (*c*) Reproduced with permission from Depan & Misra [80]. Copyright © 2012 Elsevier. (*e*) Reproduced with permission from Sanyakamdhorn *et al.* [81]. Copyright © 2013 American Chemical. Society.

The drug doxorubicin (DOX) is a highly effective chemotherapy medication. DOX comprises multiple carbonyl and hydroxyl moieties; thus, it can engage in numerous hydrogen bonds with appropriate donor/acceptor molecules (figure 12*a*). There have been a number of studies that take advantage of the hydrogen-bonding ability of DOX to bond the drug to polymeric materials. Depan and Misra demonstrated a hybrid material approach by crystallizing a block copolymer, poly(ethylene)-b-poly(ethylene glycol) (PE-b-PEG), on the long axis of carbon nanotubes (CNTs) (figure 12*b*,*c*) [80]. The PEG portion of the copolymer was able to form hydrogen bonds with DOX, as evidenced by ultraviolet–visible and IR spectroscopy. DOX could be released under acidic conditions, which weakens the hydrogen bonds between PE-b-PEG and DOX. Interestingly, ultrasonic irradiation was also used to successfully release DOX from the polymer hybrids [80].

Tajmir-Riahi and co-workers used chitosan, a polysaccharide with abundant hydrogen-bonding sites, to bind DOX in polymer nanoparticles (figure 12*d*) [81]. Three different molecular weights of chitosan were used, and the 100 kDa polymer exhibited the largest drug–polymer binding constant at *ca* 2 × 10^5^ M^−1^ (determined through fluorescence spectroscopy). Molecular modelling was used to demonstrate that DOX is surrounded by several donor atoms of the chitosan residue, and the free binding energy was found to be −3.89 kcal mol^−1^ (figure 12*e*) [81].

A different hydrogen-bonding motif capable of interacting with DOX was used by Zhang, Li and co-workers [82]. Methoxy-poly(ethylene glycol)-block-poly(acrylamide-co-acrylonitrile) (mPEG-b-P(AAm-co-AN)) copolymers with different acrylamide (AAm) content were investigated (figure 12*f*) [82]. A combination of hydrophobic and hydrogen-bonding interactions contributed to the size of the polymer micelles and ability to load DOX. Polymers with higher AAm content exhibited strong hydrogen bonding between AAm units, resulting in a compact core and small micelle diameters. On the other hand, polymers with lower AAm content exhibited less self-associative hydrogen bonding between AAm units, larger diameter micelles and were able to bond with larger quantities of DOX. The release of DOX from the polymer was accomplished via heating.

**Figure 13.**
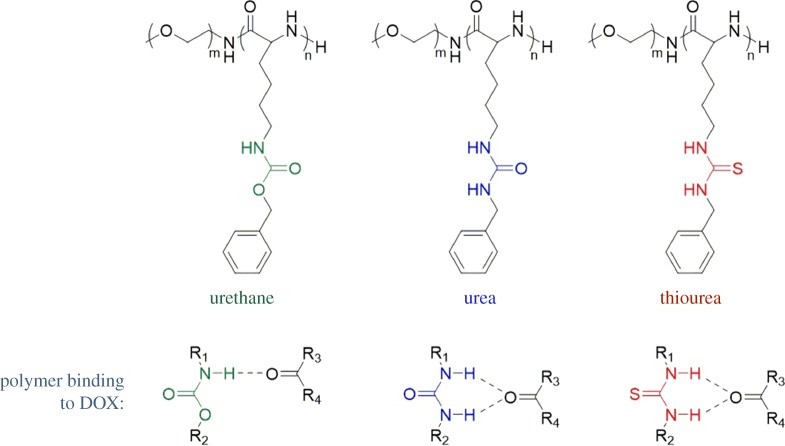
Structures of functionalized polymers and hydrogen bonds formed between the polymer motif and carbonyl group of DOX.

Recently, Zhu, Tian and co-workers investigated the ability of DOX to form hydrogen bonds with polymers containing different hydrogen-bonding motifs, namely urethane, urea and thiourea [83]. It was expected that the urethane motif could only form one-point hydrogen bonds with a carbonyl motif of DOX, while the urea and thiourea functionalities could form stronger, two-point hydrogen bonds with DOX (figure 13). Before the addition of DOX to the polymer, the size of the polymer micelles could be related to the degree of self-associative hydrogen bonding between the motifs. The diameters of the urea polymer micelles were the smallest due to strong hydrogen-bonding interactions between urea groups, while the thiourea polymer micelles were the largest in diameter due to a lack of hydrogen bonds between thiourea motifs. The loading efficiency of DOX into the urea and thiourea polymers was much higher than the urethane polymer, due to stronger hydrogen-bonding interactions between the polymer and DOX. The release of DOX from the polymers was accomplished by using near-infrared laser irradiation to destabilize the strong hydrogen-bonding interactions between the polymer and DOX.

### 4.2. Polymers that engage small biomolecules

**Figure 14.**
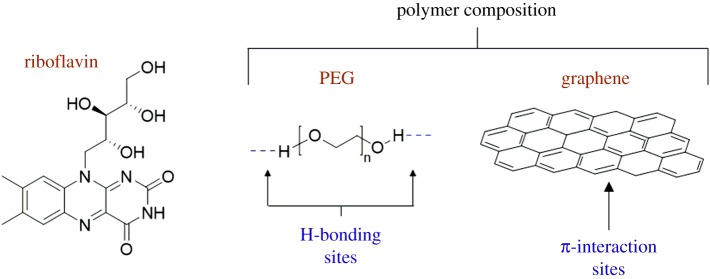
Structures of riboflavin and the polymer components, highlighting the bonding sites for riboflavin.

In addition to bonding with drug molecules, functionalized polymers have also been used to engage in hydrogen bonds with biomolecules. Riboflavin (vitamin B_2_) is a vitamin and used as a dietary supplement. The molecule itself is fluorescent, and it exhibits multiple hydrogen-bond donor and acceptor sites, (figure 14). Díez-Pascual and co-workers demonstrated the first use of non-covalent interactions between riboflavin and graphene/PEG to detect the vitamin optically (figure 14) [84]. The fluorescence of riboflavin was quenched upon interaction with the polymer. The quenching was attributed to a combination of hydrogen bonds between riboflavin and PEG and π–π stacking interactions between the riboflavin aromatic rings and π-cloud of graphene. These non-covalent interactions were successfully used to detect riboflavin in commercial vitamins, and the method shows potential for application to the optical detection of other vitamins.

**Figure 15.**
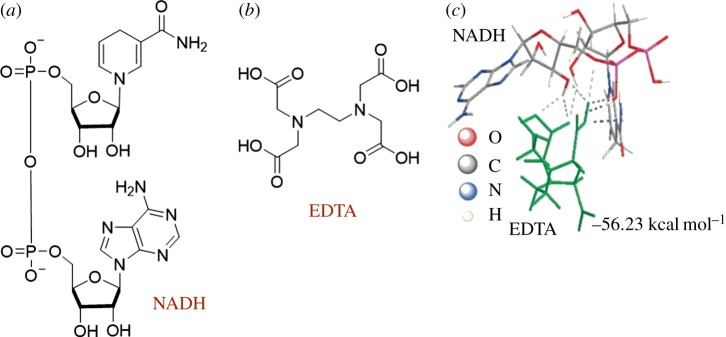
Chemical structures of: (*a*) NADH, (*b*) EDTA and (*c*) EDTA–NADH hydrogen-bonded complex. (*c*) Reproduced with permission from Akhtar *et al.* [85]. Copyright © 2016 Elsevier.

The biomolecule β-nicotinamide adenine dinucleotide (NADH) is found in all living cells, and it acts as a coenzyme in redox reactions. NADH contains an adenine base and a NICO unit (figure 15*a*). Shim and co-workers designed and constructed a sensor for complexing NADH via hydrogen bonds [85]. Ethylenediaminetetraacetic acid (EDTA) (figure 15*b*) was covalently linked to a polyethylenimine (PEI)/activated graphene oxide composite by forming an amide bond between the NH_2_ groups of PEI and the carboxylic acid groups of EDTA. The EDTA moieties formed hydrogen bonds with NADH as evidenced by cyclic voltammetry, quartz crystal microbalance and X-ray photoelectron spectroscopy measurements. Calculations of the interaction energies between EDTA and NADH were also performed. The lowest energy conformation shows three hydrogen bonds involving two carboxylic acid groups of EDTA with two hydroxyl groups and a phosphate group of NADH (figure 15*c*) [85].

**Figure 16.**
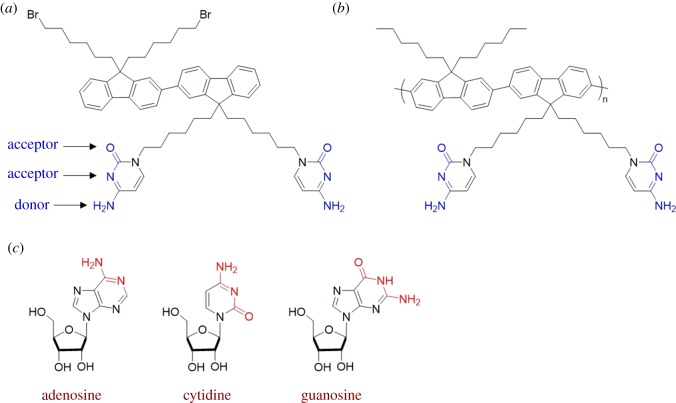
Chemical structures of: (*a*) cytosine oligomer, (*b*) cytosine polymer and (*c*) nucleosides.

Similar to the multi-point hydrogen bonding of methotrexate (§4.1), sensing of nucleosides can also be accomplished by using appropriately designed motifs. Yamaguchi and Miyawaki designed and synthesized conjugated oligomers and polymers with N_1_-hexylcytosine side chains (figure 16*a*,*b*) [86]. The cytosine motif has two hydrogen-bond acceptor sites and one hydrogen-bond donor site. The cytosine functional group is capable of engaging in hydrogen bonds with the nucleosides adenosine, cytidine and guanosine (figure 16*c*). The conjugated cytosine-functionalized polymers are photoluminescent in solution; the photoluminescence intensity decreased upon addition of the nucleosides, which indicated a quenching mechanism [86]. The guanosine nucleoside was the most effective photoluminescence quencher because it is fully complementary and can engage in three hydrogen bonds with cytosine. The adenosine and cytidine nucleosides, however, exhibit one site that is not compatible with cytosine. Thus, adenosine and cytidine can only engage in two hydrogen bonds with the cytosine-functionalized materials.

## 5. Outlook and conclusion

This review presents an overview of some synthetic approaches that can be used to design small molecules and polymers capable of forming hydrogen bonds with pharmaceuticals and small biomolecules. Many of these pharmaceuticals and biomolecules are composed of numerous hydrogen-bond donor and acceptor sites. These sites can be used to design donor/acceptor motifs that will interact with the small molecules in a highly specific manner. However, due to the variety of hydrogen bonds that the pharmaceutical is capable of forming, prediction of the intermolecular interactions, especially in crystalline systems, still needs further development. Studies that investigate the behaviour of these pharmaceuticals with multiple donor/acceptor sites in the presence of diverse recognition motifs will aid in the understanding and predictability of synthon formation for these novel, intriguing systems. For polymeric materials, recognition of small molecules via hydrogen bonding has been accomplished by using complementary, geometrically matched receptors and functional groups. Polymers that can engage in non-specific hydrogen-bonding interactions have also been successful in recognition applications. The diversity in types of molecules and motifs for recognizing small molecules is consistently increasing. Further development of chemically unique scaffolds that can sense and bond pharmaceuticals or biomolecules with reversibility will advance the fields of sensing, remediation and delivery towards truly functional materials.

## Data accessibility

This article has no additional data.
